# A Word on Words in Words: How Do Embedded Words Affect Reading?

**DOI:** 10.5334/joc.45

**Published:** 2018-09-25

**Authors:** Joshua Snell, Jonathan Grainger, Mathieu Declerck

**Affiliations:** 1 Laboratoire de Psychologie Cognitive, CNRS & Aix-Marseille Université, Marseille, FR

**Keywords:** Reading, Visual word processing, Eye movements

## Abstract

A surprisingly small portion of reading research has been dedicated to investigating how the visual word recognition process is influenced by embedded words (e.g., ‘*arm*’ in ‘*charm*’), and no research has yet investigated embedded words in a natural reading setting. Covering this issue, the present work reports analyses of eye-tracking data from the GECO bilingual book reading corpus. Word viewing times were analyzed as a function of the number, frequency and proportional length of embedded words. We anticipated two scenarios: embedded words would either facilitate processing due to increased word-letter feedback, or inhibit processing due to increased lexical competition. A main facilitatory effect of embedded words on the recognition process was established, with an increasing number of embedded words resulting in shorter word viewing times and fewer fixations. This pattern was depicted by readers of Dutch as well as readers of English. Long, high-frequency embedded words formed an exception however, as these led to inhibition (Dutch participants) or a null-effect (English participants). The present results indicate that both scenarios outlined above are at play, but with a theoretical constraint on the role of word-to-word inhibitory connections. Specifically, such connections may predominantly exist among words of similar length. Hence, embedded words generally facilitate processing through word-letter feedback, but this facilitatory effect is countered by word-to-word inhibition if the embedded word’s length approximates that of its superset.

## 1. Introduction

A primary goal of reading research has been to determine how recognition of letters leads to activation and recognition of word representations in the brain. Much at the heart of the endeavor of ‘cracking the orthographic code’ (Grainger, 2008) lies the principle that the recognition of words is influenced by other words that are either in temporal or spatial proximity. This principle has been pivotal in two ways. Firstly, observing how characteristics of one word influence the other has allowed researchers to deduce key aspects of the word recognition process—especially with respect to the encoding of letter identity and position (e.g. Snell, Bertrand & Grainger, 2018c). Secondly, word-to-word influences per se can be regarded as an intrinsic part of the word recognition process; that is, a complete understanding of how words are recognized obligates understanding of why and how words are influenced by other words. This will be the central theme of the present paper. Specifically, we cover how the recognition of words is impacted by words that are in the uttermost spatial and temporal proximity: namely, embedded words (e.g., ‘*arm*’ in ‘*charm*’).

Having been addressed in but a handful of studies,^1^ many issues concerning reading embedded words remain open to inquiry (Bowers, Davis & Hanley, 2005; Davis & Taft, 2005; Davis, Perea & Acha, 2009). The most obvious question is whether embedded words are lexically accessed at all (as noted in the title of Bowers, Davis and Hanley’s (2005) study: “*Is there a ‘hat’ in ‘that’?*”). In their study, Bowers et al. found that semantic categorization decisions about target words (e.g., ‘*Does* hatch *refer to a piece of clothing?*’) were slower and less accurate if the embedded word (‘*hat*’) was associated with a different response than the target (‘*hatch*’), hence suggesting that representations associated with embedded words may indeed be activated—and semantically processed—in parallel with representations associated with the target word. It is further worth noting that within the closely related research domain investigating morphological processing, evidence has been found for parallel processing of affixes, stems and suffixes (e.g., ‘*de*’, ‘*brief*’ and ‘*ing*’ in ‘*debriefing*’), and even so in the case of pseudo-derived words (e.g., ‘*corner*’, which is not a derivation of ‘*corn*’), thus again arguing for embedded word activation (see Grainger & Beyersmann, 2017, for a review).

The next question is what effect embedded words may have on target word processing. Note that the work of Bowers et al. (2005) does not evidence an influence of embedded words on semantic processing of the target: after all, semantic processing of the target and embedded word may have proceeded independently, with the embedded word’s semantic category only impacting at the stage of decision-making.

Concerning the embedded word’s influence on target processing, four scenarios are conceivable: either the embedded word (i) facilitates processing of the target, (ii) inhibits processing of the target, (iii) does both, potentially resulting in a null-effect, or (iv) does neither, hence certainly resulting in a null-effect. Below, we will explain why embedded words may have a facilitatory and/or inhibitory influence on processing of the word by which they are contained (their so-called *supersets*). In doing so, we will firstly draw a critical analogy to *orthographic neighbors*. Secondly, we will address the potential role of morphological relationships between embedded words and their supersets.

### 1.1. Orthographic neighbors versus embedded words

By the classic definition, orthographic neighbors are words that are identical to one another in all but one letter (e.g., ‘*rock*’ and ‘*rack*’). They play a key role in neighborhood size effects, whereby words that have a larger amount of orthographic neighbors (e.g., ‘*ball*’, ‘*bill*’, ‘*hall*’, ‘*hill*’) are recognized faster than words with a smaller neighborhood size (Andrews, 1989; Forster & Shen, 1996; Johnson & Pugh, 1994). Accounting for this effect is the conception that letter nodes are connected to the nodes of all words in which the letter occurs (such that the ‘*r*’ in ‘*rock*’ would not only activate ‘*rock*’ but also ‘*rocket*’, ‘*rack*’, ‘*race*’, et cetera), and that word nodes would in turn provide feedback activation of letter nodes (e.g., McClelland & Rumelhart 1981). Viewing a word with a large neighborhood size results in letter detectors receiving feedback activation from a higher amount of strongly activated word nodes, in turn leading to faster recognition (but see Grainger & Jacobs, 1996, for an alternative account expressed in terms of decision-level processes).

Orthographic neighbors have also been shown to facilitate processing when being presented next to one another in the visual field. In sentence reading, Snell, Vitu and Grainger (2017a) found that a target word at position *n* was recognized faster (evidenced by shorter word viewing times) if an orthographic neighbor was presented at position *n* + 1, compared to if an unrelated word of the same length and frequency was presented at that location. In a flanker paradigm wherein participants made lexical decisions about foveal target words that were flanked by words on the left and right, Snell, Bertrand, Meeter and Grainger (2018a) found that responses were faster and more accurate with neighbor flankers, compared to unrelated flankers.

It should be noted however, that orthographic neighbors have also been found to slow down, rather than speed up, word recognition. Using a masked-priming lexical decision task, Grainger & Segui (1990) found that processing of target words (e.g., ‘*rack*’) was inhibited if those targets were preceded by briefly presented higher-frequency neighbor prime words (‘*rock*’), compared to unrelated prime words of the same frequency (‘*step*’) (see also de Moor & Brysbaert, 2000; Davis & Lupker, 2006). This has led researchers to argue for the presence of inhibitory connections among word nodes—the idea being that co-active words would have to compete for recognition (e.g., Grainger & Segui, 1990; Grainger, 2008). It stands to question, then, why neighbors facilitate processing when they are spatially adjacent to the target, as has been shown in the studies of Snell et al. (2017a, 2018a). The answer offered by Snell et al. (2018a) is that multiple lexical representations may be processed in parallel, and that mutual inhibition would only occur between words that are associated with the same spatial location. Indeed, whereas Snell et al. established a facilitatory effect of neighbor flankers, they also observed an inhibitory effect when the same stimuli were used as masked primes at the target location.

Clearly, how an embedded word relates to its superset is to some extent similar to how a word relates to its orthographic neighbor. Like orthographic neighbors, embedded words and their supersets are orthographically related, but are represented by different word nodes. One might consequently expect that the embedded word should influence processing of its superset similarly to how an orthographic neighbor would.

The central problem here, then, is that it is unclear to what extent influences of embedded words should mimic neighborhood size- and neighbor flanker effects (facilitation) or masked neighbor priming effects (inhibition). On the one hand one may argue that words should be recognized faster upon the presence of an embedded word, given that the embedded word contributes to the orthographic neighborhood size of its superset, hence leading to stronger feedback activation of letters. On the other hand, it is possible that the word-to-word inhibitory connections that are postulated to exist among orthographic neighbors, also exist between embedded words and their supersets.

The few studies that have directly investigated how embedded words may influence visual word recognition, seem to argue for the latter. Investigating English and Spanish reading respectively, Davis and Taft (2005) and Davis et al. (2009) found that high-frequency embedded words slowed down responses in a lexical decision task—a finding reminiscent of the inhibitory effects of orthographic neighbors in lexical decision-making (e.g., Grainger & Segui, 1990; de Moor & Brysbaert, 2000; Davis & Lupker, 2006).

However, several uncertainties remain. For one, the inhibitory effects reported by Davis and colleagues (2005, 2009) were found with the exclusive use of low-frequency targets that contained high-frequency embedded words (e.g., ‘*come*’ in ‘*comet*’). It is possible that different behavioral patterns emerge when the respective frequencies of targets and embedded words change, given that the strength of lateral inhibition depends on the word’s initial activation level, which in turn is influenced by its frequency. A low-frequency neighbor is not likely to be strongly activated, and might therefore not yield an observable inhibitory effect. Additionally, the embedded words in aforementioned studies were always equal to the target minus one letter (coined ‘deletion neighbors’ by Davis et al.). Snell, Van Leipsig, Grainger and Meeter (2018b) have proposed that lateral inhibitory connections may exclusively exist among words of a sufficiently similar length,^2^ suggesting that while embedded words covering almost the entire superset would exert inhibition, shorter embedded words could instead facilitate due to the presence of word-to-letter feedback but absence of an inhibitory connection between the embedded word and its superset. The justification of this assumption is that if an embedded word is considerably shorter than its superset, such word length information should dispel any ambiguity that would otherwise invoke the need for a lexical competition mechanism (see Snell et al., 2018b, for the implementation of this principle in a computational model).

### 1.2. Morphemes versus embedded words

Yet another factor that may influence the embedded word’s impact on recognition of its superset, is the morphological relationship between these words. While orthographic neighbors presumably exert mutual inhibition, morphologically related words (e.g., ‘*farming*’ and ‘*farmer*’) have been theorized to activate one another through feedback from morpho-semantic nodes (‘*farm*’) that are connected to all members of the morphological family. The principle, shared by contemporary frameworks of morphological processing in reading, is that activation of the orthographic representation ‘*farming*’ would lead to activation of the morpho-semantic node ‘*farm*’, which would in turn provide feedback activation of the orthographic representation ‘*farmer*’ (Beyersmann, Castles & Coltheart, 2012; Crepaldi, Rastle, Coltheart & Nickels, 2010; Diependaele, Sandra & Grainger, 2009; Giraudo & Grainger, 2001; Grainger & Beyersmann, 2017).

Evidence for such a mechanism, mostly obtained with the masked priming lexical decision task, is mixed. On the one hand, lexical decisions about target words were made faster and more accurately when targets were preceded by morphologically related primes (e.g., ‘*teacher*’ – ‘*teach*’) than unrelated primes (‘*finally*’ – ‘*teach*’), while no such effects were observed when comparing non-morphological primes (e.g., ‘*dialog*’ – ‘*dial*’) to unrelated primes (Rastle, Davis & New, 2004; Longtin, Segui & Hallé, 2003). On the other hand, similar priming effects were reported for pseudo-derived primes that clearly do not belong to the same morphological family as the target (e.g., ‘*number*’ – ‘*numb*’), hence indicating that there must be additional mechanisms at play.

The masked priming paradigm taken aside, the literature to date is scarce as well as equivocal about facilitation from morphemes. Testing single word reading, Carlisle and Stone (2005) found that elementary school students read bi-morphemic words (e.g., ‘*shady*’) faster than mono-morphemic words (e.g., ‘*lady*’). Burani, Marcolini, De Luca and Zoccolotti (2008) obtained similar results with dyslexic children, but established no such effect with skilled children and adults. On the other hand, in a single-word lexical decision task (without primes), Hasenäcker, Schröter and Schroeder (2017) observed facilitation from compound, prefixed and suffixed words when compared to mono-morphemic words in both children and adults. Finally, in sentence reading, Niswander, Pollatsek and Rayner (2000) found that words comprising a high-frequency stem were read faster than words comprising a low-frequency stem (whole-word frequency being equal); however, to our knowledge, no sentence reading research to date has investigated the effects of the presence of stems per se.

### 1.3. The present study

Summing up the previous sections, embedded words might either have a facilitatory or inhibitory impact, depending on multiple factors. In particular, the scarce literature on this issue has suggested that long, high-frequency embedded words may inhibit processing of low-frequency supersets (Davis & Taft, 2005; Davis et al., 2009). It is unknown whether such effects hold for embedded words that are shorter and/or of higher frequency. Further, morphemes might facilitate processing, although evidence for this is largely restricted to the realm of masked priming lexical decision-making, with facilitation from pseudo-derived morphemes (‘*corner*’ – ‘*corn*’) raising doubts about whether these effects are truly morphological in nature (Rastle et al., 2004; Longtin et al., 2003).

It is clear that the field lacks thorough investigations into the impact of embedded words. Of particular concern here, is the fact that most of the aforementioned studies employed highly artificial reading settings. Prior research has shown that discrepancies between such settings and more natural reading may lead to quite different behavioral patterns and, as a potential consequence, contrasting theories (e.g., Snell, Meeter & Grainger, 2017b; Snell, Declerck & Grainger, 2017c). To our knowledge, no prior research has provided a direct test of the impact of embedded words, morphologically related to the superset or not, in sentence reading.^3^ While Davis et al. (2009) have reported a sentence reading experiment comparing targets with deletion neighbors versus targets without deletion neighbors, this experiment used targets for which the neighbor required deletion of an inner-letter (e.g., ‘*house*’, which contains ‘*hose*’), meaning that they did not test true intact embedded words (comprised of contiguous letter combinations).

Hence, attempting to draw the bigger picture concerning embedded words, the present paper assesses embedded words in a natural reading setting. This investigation invokes analyses of the GECO book reading corpus (Cop, Dirix, Drieghe & Duyck, 2017), which contains eye-tracking data of 18 Dutch-English bilingual subjects and 14 English monolingual subjects reading an entire novel.^4^ The eye-tracking data comprise a multitude of variables, including, for instance, word viewing times, which are used as a measure of word recognition speed (e.g., Rayner, 1998).

Importantly, the vast size of the GECO corpus provides us abundant statistical power to determine whether and how effects of embedded words are modulated by factors such as length and frequency. Following the proposition of Snell et al. (2018b), we hypothesize that embedded words will only inhibit processing of their superset if they are of a sufficiently similar length (alongside the criterion of having a higher frequency than the superset, in line with aforementioned studies). Embedded words that are considerably shorter than their superset should thus lead to facilitation, due to feedback activation of letters but absence of inhibitory word-to-word connections. Lastly, as outlined in Section 2.2.3, we will carry out a preliminary investigation into the role of morphology, the exploratory nature of which prevents us to formulate concrete hypotheses.

## 2. Analysis of the GECO corpus

In their study, Cop et al. (2017) let 18 Dutch-English bilingual subjects^5^ (F = 16, age range = 18–24) read the novel *The mysterious affair at Styles*, by Agatha Christie. This novel was chosen for being freely accessible on the internet in many different languages (allowing for replication in other languages) as well as having a below-average reading difficulty (Cop et al., 2017). The novel was read both in Dutch and in English, with 9 subjects reading the first half of the book in Dutch and the second half in English, while the 9 other subjects read the first half in English and the second half in Dutch. Language proficiency tests pointed out that the subjects’ L2 proficiency was of an upper-intermediate (60–80%) level. Additionally, the authors tested 14 English monolinguals (F = 8, age range = 18–36) whom read the whole book in English.

Eye movement data was collected for 59,716 words in the Dutch version of the novel (per two subjects, as Dutch subjects read half of the book in English), and for 54,364 words in the English version. The text was presented in paragraphs (with a maximum of 145 words per display) with black 14-point Courier New font on a light grey background and with triple line spacing. Subjects could press a button on a control pad to move from one paragraph to the next. After each of the 18 book chapters, subjects were presented multiple-choice questions to ensure that they had paid attention throughout the chapter. Reading of the entire novel took approximately four hours and was carried out in four 1-hour sessions. Eye movements were tracked with the EyeLink 1000 system (SR Research, Canada) with a sampling rate of 1 kHz.

### 2.1. Our datasets

From the GECO corpus we abstracted two datasets, representing Dutch reading (by 18 Dutch bilingual subjects) and English reading (by 14 monolingual English subjects). We retrieved each subject’s first 2000 words with a length between 4 and 15 characters, amounting to a total of 36,000 datapoints for Dutch reading and 28,000 datapoints for English reading. Names (e.g., ‘*John*’) and contractions (e.g., ‘*don’t*’) were avoided.

For each datapoint we determined the set of embedded words contained by the target word. This was done by checking for each of the target’s contiguous letter combinations (e.g., ‘*ro*’, ‘*oc*’, ‘*ck*’, ‘*roc*’ and ‘*ock*’ in ‘*rock*’) whether it occurred in the Dutch Lexicon Project database of Keuleers, Diependaele and Brysbaert (2010) for Dutch words, or the British equivalent by Keuleers, Lacey, Rastle and Brysbaert (2012) for English words. The resulting number of embedded words was one of the independent variables used in our analyses. We further determined the length of embedded words relative to the target word, by dividing the average embedded word length by the target word length. Embedded word frequency was calculated by averaging the embedded words’ log-transformed book frequencies (as reported in Keuleers et al., 2010, 2012).

We also marked items that exclusively comprised edge-aligned embedded words, as well as items that exclusively comprised non-edge-aligned embedded words (e.g., ‘*or*’ in ‘*word*’). This was done to inspect the potential role of morphological relationships between embedded words and their supersets, with the rationale that edge-aligned embedded words are considerably more often morphemes (e.g., ‘*farm*’ in ‘*farmer*’) than non-edge-aligned embedded words (Grainger & Beyersmann, 2017).

Finally, for each target word we retrieved the gaze duration (GD) and the total viewing time (TVT) from the GECO corpus. GD reflects the sum of all first-pass fixation durations on a word, and arguably provides the most direct measure of word recognition speed (e.g., Rayner, 1998). TVT represents the sum of all fixation durations on a word (that is, including fixations following a regression), and reflects contextual comprehension. We further determined how many times each target was fixated (with zero times indicating a word skip).

**Figure 1:**
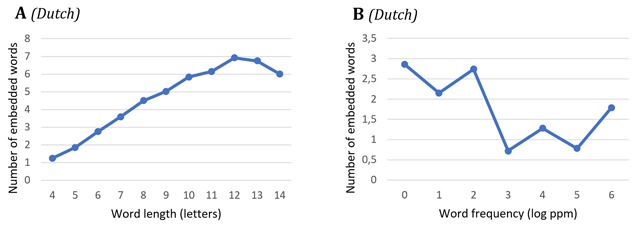
Descriptive statistics of the average number of embedded words as a function of word length **(A)** and word frequency **(B)** in Dutch reading.

**Figure 2:**
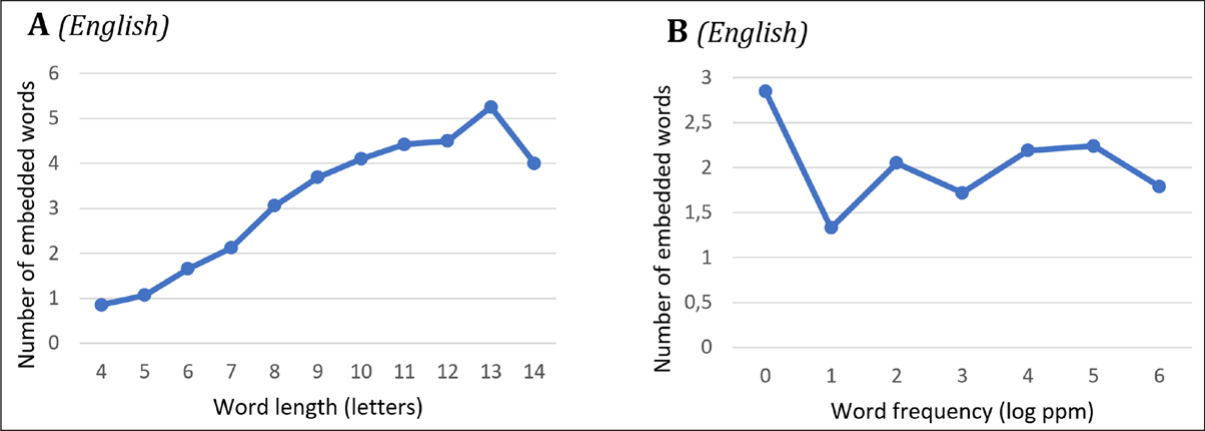
Descriptive statistics of the average number of embedded words as a function of word length **(A)** and word frequency **(B)** in English reading.

**Figure 3:**
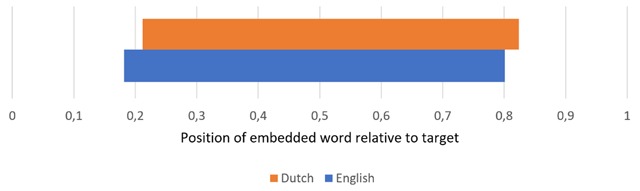
Average position of embedded words relative to the target, with the target’s left and right boundary corresponding to 0 and 1 respectively.

Some descriptive statistics about our datasets are shown in Figures 1, 2, 3. Unsurprisingly, longer targets contain more embedded words on average (which is why target length was added as a factor in our models; Section 2.2.1). The number of embedded words does not go up with target frequency. In Dutch targets, the position of embedded words is on average slightly shifted to the right (by about 2–3%) compared to English targets (Figure 3).

### 2.2. Results

Below, our analyses are presented separately for Dutch and English reading. Skipped words (33.2%) were excluded from the word viewing time analyses. Words with a word viewing time (as reflected in GD) beyond 2.5 SD from the grand mean (1.81% of words) were excluded from the analyses of word viewing times as well as number of fixations.

We employed linear mixed-effect models (LMMs) with subjects entered as random factor (e.g., Baayen, 2008).^6^ The models were fitted with the lmer function from the lme4 package (Bates, Maechle, Bolker & Walker, 2015) in the R statistical computing environment. Following Barr, Levy, Scheepers and Tily (2013) we determined the maximal random effect structure permitted by the data. This led us to include by-subject random slopes alongside the random intercept. We report *b*-values, standard errors (SEs) and *t*-values for all factors, with *t*-values of |1.96| and beyond deemed significant.

#### 2.2.1. Dutch reading

We firstly tested for a main effect of the number of embedded words on target word recognition speed. The LMM for this analysis included the number of L1 embedded words and target word length as factors. The latter variable was included to account for its cofound with the number of embedded words: i.e., longer words, which are usually recognized slower,^7^ are likely to contain more embedded words (Figure 1A).

**Figure 4:**
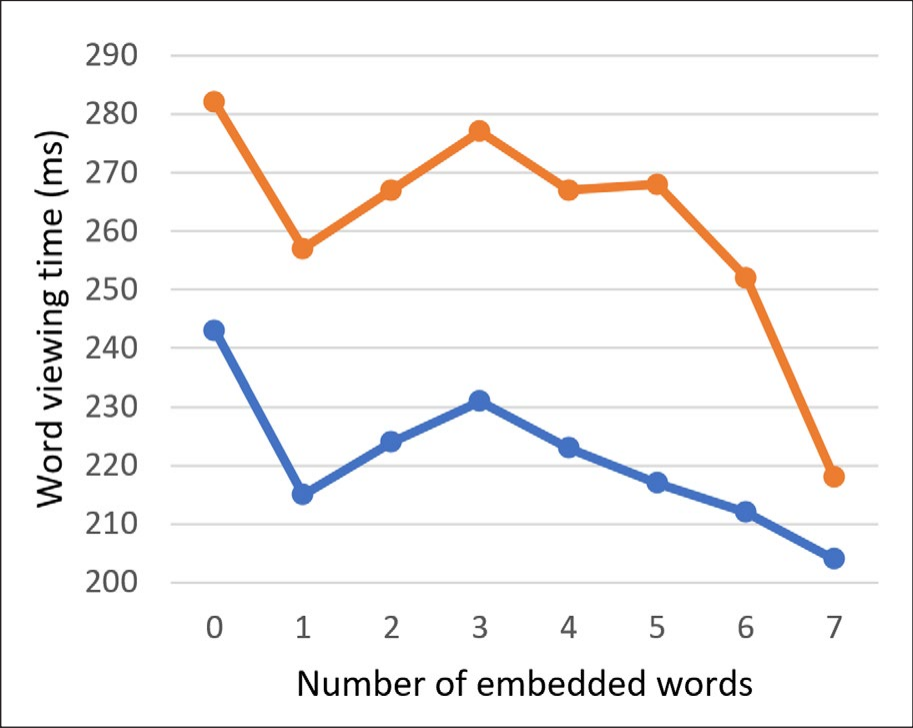
Word viewing times (gaze duration (GD) and total viewing time (TVT)) as a function of the number of embedded words in 6-letter Dutch targets.

As it turned out, word viewing times decreased as the number of embedded words increased: in GD, *b* = –2.93, SE = 0.79, *t* = –3.71; in TVT, *b* = –4.91, SE = 1.48, *t* = –3.31 (see word viewing times on 6-letter words in Figure 4). Significantly fewer fixations were made upon an increasing number of embedded words: *b* = –0.03, SE = 0.01, *t* = –4.46. We established an interaction of the number of embedded words and target word length, with the effect of embedded words on GD and number of fixations being more strongly pronounced in longer target words: in GD, *b* = 0.37, SE = 0.07, *t* = 5.31; in TVT, *b* = 0.45, SE = 0.12, *t* = 3.80; in number of fixations, *b* = 2.49 * 10^–3^, SE = 5.30 * 10^–4^, *t* = 4.70.

Next, we assessed whether the effect of embedded words on target word recognition was modulated by embedded word length. This was done by including embedded word length as a variable in the model alongside number of embedded words and target word length. A significant interaction of embedded word length and the number of embedded words on word viewing times was established, with *b* = 12.02, SE = 5.19, *t* = 2.32 in GD, and *b* = 27.54, SE = 9.92, *t* = 2.78 in TVT. This interaction was also expressed in the number of fixations: *b* = 0.09, SE = 0.04, *t* = 2.16. Aiming to explore the nature of this interaction, we split the dataset in two, based on embedded word length being above or below the median of 0.46 (this is a normalized value with 1 corresponding to the target word length). Crucially, when running the original model on these two datasets, we found that relatively short embedded words facilitated target processing (GD: *b* = –4.70, SE = 0.95, *t* = –4.94; TVT: *b* = –6.28, SE = 1.89, *t* = –3.32; number of fixations: *b* = –0.03, SE = 0.01, *t* = –4.84) whereas no effect was found for the relatively longer embedded words (GD: *b* = 1.70, SE = 1.81, *t* = 0.94; TVT: *b* = 2.09, SE = 3.15, *t* = 0.67; number of fixations: *b* = –0.01, SE = 0.01, *t* = –0.69).

Our assessment of embedded word frequency followed the same procedure. Here, a marginally significant interaction was established between embedded word frequency and the number of embedded words, (GD: *b* = 1.18, SE = 0.66, *t* = 1.78; TVT: *b* = 2.01, SE = 1.17, *t* = 1.71; number of fixations: *b* = 1.18, SE = 0.66, *t* = 1.78), such that the facilitatory main effect of embedded words was slightly stronger when they were low-frequency (i.e., below the frequency median of 4.95: *b* = –3.60, SE = 0.94, *t* = –3.84) compared to when they were high-frequency (i.e., above the frequency median: *b* = –2.09, SE = 1.11, *t* = –1.88). Considering that prior single word reading research has only established inhibitory effects using high-frequency embedded words (Davis & Taft, 2005; Davis et al., 2009), we further scrutinized the subset of data with long embedded words for which we found no significant effect. Aligning with the observations of Davis and colleagues, an inhibitory effect emerged with long embedded words above the frequency median (GD: *b* = 9.74, SE = 3.16, *t* = 3.08; TVT: *b* = 13.75, SE = 4.69, *t* = 2.93; number of fixations: *b* = 0.10, SE = 0.02, *t* = 3.92) while no effect was observed for long embedded words below the frequency median (*b* = –0.79, SE = 2.37, *t* = –0.33; TVT: *b* = –5.61, SE = 4.26, *t* = –1.32; number of fixations: *b* = 0.04, SE = 0.02, *t* = 1.44).

#### 2.2.2. English reading

**Figure 5:**
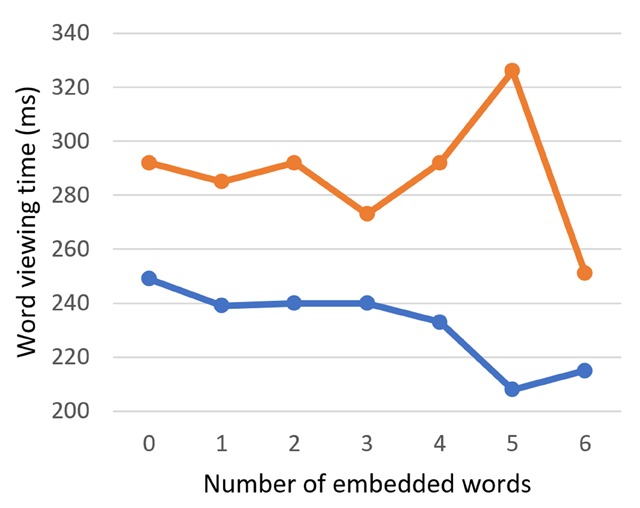
Word viewing times as a function of the number of embedded words in 6-letter English targets, (one might note that the facilitatory effect of embedded words on TVT, as reported in the text, is not apparent in this figure; this is likely because the facilitatory effect is driven by targets of other lengths).

Like the Dutch-English bilingual readers, the English monolingual readers showed a facilitatory main effect of the number of embedded words on the word viewing time (GD: *b* = –2.00, SE = 1.16, *t* = –1.72; TVT: *b* = –6.07, SE = 1.95, *t* = –3.12) and number of fixations (*b* = –0.02, SE = 0.01, *t* = –2.05) (Figure 5). The number of embedded words again interacted with target word length (GD: *b* = 0.27, SE = 0.14, *t* = 2.02; TVT: *b* = 0.78, SE = 0.23, *t* = 3.43; number of fixations: *b* = 3.70 * 10^–3^, SE = 1.25 * 10^–3^, *t* = 2.95).

However, the English monolinguals did not show an interaction between number of embedded words and embedded word length (GD: *b* = –3.69, SE = 55.50, *t* = –0.07; TVT: *b* = –22.63; SE = 13.57; *t* = –1.67; number of fixations: *b* = 0.08, SE = 0.07, *t* = 1.13) or embedded word frequency (GD: *b* = 2.69, SE = 3.22, *t* = 0.83; TVT: *b* = 1.58, SE = 1.09, *t* = 1.45; number of fixations: 3.81 * 10^–3^, SE = 0.01, *t* = 0.40). Indeed, this time around, no inhibition of embedded words was observed for cases above the length- and frequency medians (0.50 and 4.48 respectively), with *b* = –2.46, SE = 5.52, *t* = –0.45 for GD; *b* = 5.39, SE = 6.03, *t* = 0.90 for TVT; and *b* = –0.02, SE = 0.03, *t* = –0.61 for the number of fixations.

#### 2.2.3. Morphology

Lastly, we assessed whether the main facilitatory effect of number of embedded words may have been driven by morphological relationships between the embedded words and their supersets. Given the obvious impracticality of manually determining morphological relationships between all items and each of their respective embedded words (necessitating one to evaluate more than 175,000 word pairs within the present datasets), we opted for an alternative strategy whereby we made use of the fact that stems (e.g., ‘*farm*’ in ‘*farmer*’) are most often edge-aligned (Grainger & Beyersman, 2017). We thus reasoned that, if the facilitatory effects were driven by morphemes, these effects should be stronger when isolating items that exclusively comprise edge-aligned embedded words.^8^

As it turned out, in Dutch reading, no influence of number of embedded words was observed in the subset of items with exclusively edge-aligned embedded words (56.49% of all items): in GD, *b* = –0.85, SE = 3.15, *t* = –0.27; in TVT, *b* = –1.16, SE = 4.66, *t* = –0.25; in number of fixations, *b* = 0.03, SE = 0.02, *t* = 1.35. On the other hand, inhibition was found in items that exclusively contained non-edge-aligned embedded words (17.25% of all items), albeit exclusively in the GD measure: in GD, *b* = 12.26, SE = 5.77, *t* = 2.13; in TVT, *b* = –3.87, SE = 8.13, *t* = –0.48; in number of fixations, *b* = –0.02, SE = 0.05, *t* = –0.53.

In the English dataset, edge-aligned embedded words (53.20% of all items) were found to facilitate: in GD, *b* = –18.52, SE = 5.26, *t* = –3.52; in TVT, *b* = –10.60, SE = 6.00, *t* = –1.77; in number of fixations, *b* = –0.05, SE = 0.03, *t* = –1.82. No effects were observed in items exclusively containing non-edge-aligned embedded words (26.00% of all items): in GD, *b* = 4.02, SE = 5.70, *t* = 0.71; in TVT, *b* = –5.46, SE = 8.35, *t* = –0.65; in number of fixations, *b* = –0.03, SE = 0.05, *t* = –0.68.

## 3. Discussion

Through decades of reading research, the question of whether and how embedded words might influence the visual word recognition process has received relatively little attention. Yet, the investigation of embedded words may provide a valuable contribution to our understanding of the reading process, given that observations of facilitation or inhibition from embedded words would be informative about the existence of word-to-letter feedback connections and word-to-word inhibitory connections in the brain. Concretely, if embedded words were found to speed up the recognition process, this would provide further evidence for word-to-letter feedback, with the rationale that a stimulus comprising more embedded words would lead to the activation of more word nodes. Those word nodes in turn provide more feedback activity to letter nodes, leading to faster word recognition (cf. the orthographic neighborhood size effect; e.g., Andrews, 1989; Forster & Shen, 1996; Johnson & Pugh, 1994). Alternatively, if embedded words were found to inhibit the recognition process, this would support the idea that word-to-word inhibitory connections exist among lexical competitors (e.g., Grainger & Segui, 1990; de Moor & Brysbaert, 2000; Davis & Lupker, 2006).

Our analyses show that, in Dutch reading, embedded words can have both a facilitatory and inhibitory impact on target word recognition: that is, when embedded words are short (less than half the target’s length), they tend to speed up recognition of the target. In contrast, longer embedded words can slow down the recognition process, on the premise that they are of high frequency. These results reaffirm earlier claims that embedded words may inhibit the recognition process (Davis & Taft, 2005; Davis et al., 2009), although it should be noted that, overall, embedded words rather tend to facilitate the recognition process (as illustrated by a facilitatory main effect of the number of embedded words).

Strikingly, in English reading, no inhibitory effect was observed at all, even when embedded words were long and of high frequency. This null-result contrasts with the pattern of effects in lexical decision times observed by Davis and Taft (2005) as well as with the pattern of effects depicted by Dutch subjects of the GECO corpus. These discrepancies may have been caused by cross-lingual differences (Dutch versus English reading) and the different natures of the respective tasks (lexical decision versus natural reading). With respect to task differences, for example, it is worth noting that while lexical decisions are facilitated by an increased orthographic neighborhood size, sentence reading is slowed (Pollatsek, Perea & Binder, 1999). Cross-lingual discrepancies are not uncommon either: as outlined by Andrews (1997), neighborhood frequency effects have produced more reliable inhibition in Spanish and French than in English.

Morphology is one potentially relevant factor. Prior research has shown facilitated processing of morphologically complex words (i.e., words consisting of more than one morpheme) comprising a high-frequency root or lexeme, compared to morphologically complex words comprising a low-frequency root or lexeme, in English sentence reading (Niswander et al., 2000). One might in this light wonder whether a higher proportion of target words comprised a root or lexeme in the English data compared to the Dutch data in the present analyses—which, in a more general sense, would be the result of key differences between the Dutch and English morphology. However, as shown in Section 2.2.3, the proportion of items with exclusively edge-aligned embedded words was approximately equal in Dutch and English (56.94% and 53.20% of all items, respectively). Hence, although practical constraints prevented us from obtaining an absolute count of the number of morphemes in both languages, we estimate that the number of morphemes must have been fairly equal. Here, then, it is noteworthy that edge-aligned embedded words, which more often consist of stems (e.g., ‘*farm*’ in ‘*farmer*’) facilitated word processing in English, while no effects of edge-aligned embedded words were observed in Dutch. This means that if morphology was a critical factor driving differences between Dutch and English reading, then such differences would be reflected in the way morphemes affect processing in each respective language, rather than in the number of morpheme occurrences per se. Naturally, such accounts of aforementioned discrepancies are at this point mere speculation, but would be worthy of further investigation in future research. Notably, future studies should ideally be able to assess the impact of morphological relationships directly, rather than through testing the influence of edge-alignedness, the indirect nature of which is a shortcoming in the present study.

Overall, the main facilitatory effect of embedded words (observed both in Dutch and English reading) puts a theoretical constraint on the role of word-to-word inhibitory connections. In line with the proposition of Snell et al. (2018b), it is possible that such connections do not simply exist among all words that share letters. Rather, a prerequisite for such connections to exist—or at least to have a considerable impact—seems to be that words must have a sufficiently similar length. Indeed, from a theoretical perspective this makes sense: word-to-word connections were theorized to function as a means to prevent that multiple words are recognized upon viewing a single word (e.g., without these connections, viewing ‘*rack*’ may lead to simultaneous recognition of ‘*rack*’ and ‘*rock*’). If an embedded word is considerably shorter than the word by which it is contained, however, such word length information should dispel any ambiguity that would otherwise drive the need for a lexical competition mechanism.

Our exploratory investigation into the role of morphology has not provided a clear answer about whether the observed facilitatory effects are driven by morphologically related embedded words. On the one hand, the inhibitory effect of non-edge-aligned embedded words (i.e., words that are presumed to be largely morphologically unrelated to the superset) in Dutch reading might be taken as evidence that the facilitatory effects must have been driven by morphemes. On the other hand, the absence of this effect in English, alongside the absence of a facilitatory effect of edge-aligned embedded words in Dutch, casts doubt on the idea that morphological relationships were the main driving force here. As it is more generally unclear how morphemes influence the word recognition process (e.g., reports of facilitation from pseudo-derived morphemes) it seems that the field of morphology research is in dire need of means to overcome these knowledge gaps before we can reasonably start disentangling effects of morphemes on the one hand, and general effects of embedded words on the other.

In sum, in the present paper we have aimed to shed some light on the impact of embedded words in natural reading. The finding that words are generally recognized faster when they contain an increased amount of embedded words supports the idea that word nodes provide feedback activation to letter nodes. However, when embedded words are long and highly frequent, the recognition process may be slowed, suggesting that word-to-word inhibitory connections may only exist among words of similar length.

## Data Availability

The corpus data is publicly available at http://expsy.ugent.be/downloads/geco/.

## Footnotes

Note that this pertains to visual word recognition specifically. Quite a few studies have investigated embedded words in spoken word recognition: see Bowers, Davis and Hanley (2005) for a discussion of these studies.

Concretely, in our model of reading, OB1 reader (Snell et al., 2018b), we have proposed that word lengths are estimated with a 0.20 error margin. As such, a 6-letter word would be sufficiently similar to a 7-letter word.

Weingartner, Juhasz and Rayner (2012) have also tested embedded words in sentence reading; however, their study did not include a baseline condition using targets without embedded words (rather, they compared targets with a high-frequency embedded word to targets with a low-frequency embedded word). This study is therefore not informative about the impact of embedded words.

The corpus data is publicly available at http://expsy.ugent.be/downloads/geco/.

Data from a 19th bilingual subject was also reported in Cop et al. (2017); however, this subject only read half of the book, and is thus not included in our analyses.

Note that LMMs for analyzing data obtained with a factorial design generally include items alongside participants as crossed random effects, to take into account the possibility that predictors exert varying effects from item to item. In the present study, however, there was no theoretical ground for adding items as random effect to the models: our variables of interest (length, frequency, number of embedded words) were already fully contingent on the identities of words, meaning that any variance within the variables is accompanied by different levels of the item factor and vice-versa. Based on this complete overlap, we had no reason to assume that the item factor accounts for additional variance in a meaningful way.

We indeed found that the fixation duration increased with word length: *b* = 5.75, SE = 0.50, *t* = 11.54.

It must be acknowledged that this approach only allows for tentative conclusions concerning the role of morphology. Complete insight into the role of morphology warrants a direct comparison of morphologically related versus unrelated embedded words.
